# Impact of an invasive nitrogen-fixing tree on arbuscular mycorrhizal fungi and the development of native species

**DOI:** 10.1093/aobpla/plw018

**Published:** 2016-03-16

**Authors:** Alejandra Guisande-Collazo, Luís González, Pablo Souza-Alonso

**Affiliations:** Department of Plant Biology and Soil Science, University of Vigo, 36310 Vigo, Spain

**Keywords:** *Acacia dealbata*, DGGE, microbial community structure, plant invasion, *Plantago lanceolata*, root inoculum, soil sterilization

## Abstract

Arbuscular mycorrhizal fungi (AMF) are obligate soil biotrophs that establish intimate relationships with 80 % of terrestrial plant families. Arbuscular mycorrhizal fungi obtain carbon from host plants and contribute to the acquisition of mineral nutrients, mainly phosphorus. The presence of invasive plants has been identified as a soil disturbance factor, often conditioning the structure and function of soil microorganisms. Despite the investigation of many aspects related to the invasion of *Acacia dealbata*, the effect produced on the structure of AMF communities has never been assessed. We hypothesize that *A. dealbata* modifies the structure of AMF community, influencing the establishment and growth of plants that are dependent on these mutualisms. To validate our hypothesis, we carried out denaturing gradient gel electrophoresis (DGGE) analysis and also grew plants of *Plantago lanceolata* in pots using roots of native shrublands or from *A. dealbata*, as inoculum of AMF. Cluster analyses from DGGE indicated an alteration in the structure of AMF communities in invaded soils. After 15 weeks, we found that plants grown in pots containing native roots presented higher stem and root growth and also produced higher biomass in comparison with plants grown with *A. dealbata* inoculum. Furthermore, plants that presented the highest biomass and growth exhibited the maximum mycorrhizal colonization and phosphorus content. Moreover, fluorescence measurements indicated that plants grown with *A. dealbata* inoculum even presented higher photosynthetic damage. Our results indicate that the presence of the invader *A. dealbata* modify the composition of the arbuscular fungal community, conditioning the establishment of native plants.

## Abstract (precis)

This study contributes to knowledge on the effect of the invasive N_2_-fixing tree, *Acacia dealbata*, on soil microbial communities and consequences on plant species that are dependent on symbiotic relationships as in the case of *Plantago lanceolata*. The main results of this work indicate that *Acacia dealbata* modifies the structure of arbuscular mycorrhizal fungi in the invaded shrublands and consequently the growth and development of plants that depend on AMF. *Plantago lanceolata* showed a substantial reduction in growth, biomass, fungal colonization and P content in the absence of native AMF species.

## Introduction

Plants cope with environmental stresses daily, without the possibility of escape, being forced to relate and extend intimate relationships with their immediate neighbours. Among them, arbuscular mycorrhizal fungi (AMF) are one of the most important symbiotic associations in nature (Willis *et al.* 2013). These plant–fungus associations form specialized interfaces, where the exchange of materials occurs between living cells (Pfeffer *et al.* 2001).

Belonging to *Glomeromycota* phylum, these fungi are usually obligated mutualists that establish intimate relationships with 80 % of terrestrial plant families (Smith and Read 1997; Brundrett 2004; Harrison 2005; Willis *et al.* 2013). The relationship can also be parasitic, depending on the combination of fungal–plant species and environmental conditions (Klironomos *et al*. 2000; Klironomos 2003). In their mutualistic associations with plants, they obtain carbon from the host plant, contributing simultaneously with the acquisition of mineral nutrients, mainly phosphorus (P) (Harrison 2005; Willis *et al.* 2013). Arbuscular mycorrhizal fungi are considered non-host plant specific (Giovannetti and Hepper 1985; Zhang *et al.* 2010), but there are studies that have shown preference between fungus and plant species (Vandenkoornhuyse *et al.* 2002; Croll *et al.* 2008). Therefore, there are plant species that completely depend on AMF associations to survive (Van der Heijden *et al.* 2008). Arbuscular mycorrhizal fungi–plant interactions, at individual or community levels, are conditioned by several factors. Abiotic conditions, such as nutrients or micro-environmental soil conditions, entail small-scale patchiness in the abundance of AMF (Mummey and Rillig 2008), and biotic factors such as competence or predation can contribute to the modification of plant–fungi associations (Lin *et al.* 2015). Among them, the presence of invasive alien plants (IAPs) has been identified as a factor altering soil communities, despite interactions between fungi and host plants (Zhang *et al.* 2010). In their relationships with AMF, successful IAPs are usually related with three criteria: nonmycorrhizal or facultative symbionts, obligated symbionts but flexible in its associations or transported together with their symbionts (Richardson *et al.* 2000; Pringle *et al*. 2009). There are many examples in the literature, which show the impact of IAPs on the structure of AMF (Richardson *et al.* 2000; Klironomos 2003; Hawkes *et al.* 2006; Broz *et al.* 2007; Vogelsang and Bever 2009; Yang *et al.* 2014).

It is well known that the presence of IAPs is usually accompanied by a reduction of native biodiversity, as they are capable of replacing native species (Richardson *et al.* 2000; Reinhart *et al.* 2005; Broz *et al.* 2007; Brewer 2008; Hoyos *et al.* 2010; de Abreu and Durigan 2011). Modifications produced can alter biotic or abiotic components of the soil environment, influencing the growth of plant species that depend on soil microorganisms (Bever *et al.* 1997). Nevertheless, due to the visual impact that the invasive species produces in the aboveground, in many cases, the belowground effects remain unexplored. There are several mechanisms that IAPs may be using to outcompete native plants and one of the most important is the direct modification of the structure and function of soil microbial communities (Richardson *et al.* 2000; Hawkes *et al.* 2006; Zhang *et al.* 2010; Tanner and Gange 2013; Souza-Alonso *et al.* 2014). *Acacia dealbata* is a leguminous tree native to Australia that has become a dangerous invader throughout the world (Richardson and Rejmánek 2011). The plant causes damage at several different levels on the invaded ecosystems, and this can include a severe decrease in native biodiversity (Fuentes-Ramírez *et al.* 2010; Lorenzo *et al.* 2010, 2012), seed bank composition (González-Muñoz *et al.* 2012), modification of decomposition processes (Castro-Díez *et al.* 2012), soil biochemical composition (Lorenzo *et al.* 2010), changes in soil microbial communities (Lorenzo *et al.* 2010) or changes in soil microbial function (Souza-Alonso *et al.* 2014). It has also been suggested that the alteration, in both soil chemistry and microbial community, is highly related to the type of ecosystem and with the age of invasion (Souza-Alonso *et al.* 2014, 2015). Despite the many aspects that have been investigated in relation with *A. dealbata* invasion, the effect produced by the entrance of the invader on the structure of native AMF communities has never been assessed. It was suggested that, at least, this species does not seem to be benefited by specific associations with AMF (Crisóstomo 2012). Therefore, we hypothesize that *A. dealbata* could change the structure of AMF community and these changes influence the establishment and growth of plants that are dependent on AMF, as in the case of *Plantago lanceolata* (fam. Plantaginaceae). Due to its AMF dependence, *P. lanceolata* is commonly used as a model species in mycorrhizal studies (Gange and West 1994; De Deyn *et al.* 2009; Cotuna *et al.* 2013; Lorenzo *et al.* 2013). This species is commonly found in the studied region associated with several ecosystems, including Atlantic shrublands.

Therefore, we have two main objectives: firstly, to compare AMF diversity in soils invaded by *A. dealbata* with non-invaded soils by using denaturing gradient gel electrophoresis (DGGE) technique, and, secondly, to evaluate the effect of changes in AMF composition on the growth of *P. lanceolata*, under controlled conditions in a greenhouse experiment.

## Methods

### Preliminary soil sampling

With the aim of evaluating the global effect of *A. dealbata* on AMF communities and justifying an extended greenhouse assay, we carried out a preliminary study of AMF structure in the invaded communities. With this objective, we sampled three separate shrublands (S_1_, 42.266397, −8.208299; S_2_, 42.305789, −8.171543; and S_3_, 42.306568, −8.172608) invaded by *A. dealbata* in O Ribeiro Region (Galicia, NW Spain). Atlantic shrublands are very common in this region and they are mainly dominated by *Ulex europaeus*, *Pterospartum tridentatum*, *Erica cinerea*, *E. umbellata*, *Calluna vulgaris*, *Anchusa* sp. and *Lotus* spp. In each shrubland, we clearly identified an invaded area (totally covered by *A. dealbata*) and a native area (without *A. dealbata* presence). Native areas were located contiguously to the invaded areas, assuring that soil characteristics were the same. At each sampling point, surface litter was removed and 30 soil samples were collected from the rhizosphere of at least 10 mature plants of *A. dealbata* (in the invaded zone) and from at least 10 mature plants of the native shrubland (a mix of the dominant species mentioned above) and pooled. Soil was immediately taken to the laboratory, freshly sieved (0.2 mm) and frozen at −20 °C until DGGE analyses were carried out.

### DNA extraction and DGGE analyses

Soil DNA extractions were performed using an UltraClean Soil DNA Isolation Kit (MO BIO Laboratories, Inc., Carlsbad, CA). An aliquot of 0.2 g soil of six replicates was used per extraction and stored at −20 °C. DNA extracted from soil samples was amplified using the primers AM1 (Helgason *et al.* 1998) and NS31-GC (Kowalchuk *et al.* 1997). All reactions were carried out in a final volume of 25 µL containing 1× polymerase chain reaction (PCR) buffer, 2.5 U Taq DNA polymerase (VWR), 0.25 mM dNTP, 0.5 µM AM1, 0.003 g bovine serum albumin, 96 % electrophoresis degree (SIGMA) and 1 µL of extracted genomic DNA. Polymerase chain reaction conditions are described in Hassan *et al.* (2013): one cycle at 94 °C for 3 min, followed by 30× (94 °C, 45 s; 58 °C, 45 s; 72 °C, 45 s) and a final extension step at 72 °C for 10 min. Polymerase chain reaction products, in aliquots of 5 µL, were analysed in 1 % agarose gel electrophoresis stained with GelRed™ and then subjected to DGGE. The PCRs were performed using a T100 Thermal Cycler (Bio-Rad, Hercules, CA, USA).

Denaturing gradient gel electrophoresis was performed with a DGGE-2401 system from CBS Scientific (San Diego, CA, USA). Twenty microlitres of each PCR product of the soil samples were analysed. Denaturing gradient gel electrophoresis analyses were conducted in 1× TAE buffer at a constant temperature of 60 °C at 20 V for 15 min, followed by 16 h at 70 V. Gels contained 8 % (w/v) acrylamide for fungi PCR products.

The linear gradient used was from 26 to 67 %, while 100 % denaturing acrylamide was defined as containing 7 M urea and 40 % (v/v) formamide. Gels were stained with 1× GelStar for 20 min and destained in distilled water for 30 min, after which they were visualized in a UV-transilluminator.

### Experimental design and sampling

After the differences found due to *A. dealbata* presence in the structure of AMF communities inferred from DGGE results, the consequences of structural changes in the growth of *P. lanceolata* were evaluated. We compared the effect of AMF from native sites (shrublands) with AMF from invaded sites using roots as inoculum (Klironomos and Hart 2002; Gu *et al.* 2011; Hassan *et al.* 2013). Mycorrhizal root fragments or active hyphal networks are both viable infection units, especially in thriving habitats (Smith and Read 2008).

To create our inoculum, soil and roots were collected in the first week of March 2013, from the same places described in the Preliminary soil sampling. In each area, two different plant materials were selected, forming the inoculum of our two treatments: from the invasive *A. dealbata* and from the dominant species of native shrubland. At the same time, soil was collected to fill pots in which *P. lanceolata* would be sown. At each sampling area, roots from at least 25 different plants of *A. dealbata* were collected using hand-scissors. Similarly, 25 different plants of shrubland species were removed with the use of a shovel, and roots carefully cut and placed in plastic bags. At the same time, 30 samples of soil were collected randomly in each zone within the first 15 cm (±1 cm) with a hand shovel and pooled together. Soil and plant material were immediately taken to the laboratory for further processing. Once in the laboratory, the soil was sieved (2 mm) and sterilized by autoclaving for three consecutive days (Nazeri *et al*. 2013). Roots from acacia and shrubland were chopped into small pieces (±1 cm) to facilitate incorporation and homogenize their distribution in the pots. After that, roots were separated into two different fractions; one part was individually sterilized together with soils and the other remained untreated. The roots were mixed with sterilized soil in plastic pots (375 cm^3^) previously sterilized (ethanol, 80 % and UV-light, 30 min) and then filled in a laminar chamber with a mixture of sterile soil : perlite : roots in a ratio of 300 : 10 : 1; perlite was added to ameliorate water retention and facilitate root development. A total of four treatments were created: (i) sterilized acacia roots (SA), (ii) non-sterilized acacia roots (NSA), (iii) sterilized shrub roots (SS) and (iv) non-sterilized shrub roots (NSS) and control without roots. The inclusion of two treatments with sterilized roots is due to the putative effect of root decomposition (*Acacia* vs. shrub) providing a different source of nutrients. The pots were arranged in a completely randomized design with nine replicates. The experimental set-up was carried out in a greenhouse at the University of Vigo (NW Spain).

In each pot, five seedlings of *P. lanceolata* were sown. This species was selected by its use as a model species in mycorrhizal studies (Gange and West 1994; Cotuna *et al.* 2013; Lorenzo *et al.* 2013). Seeds were previously sterilized in a sodium hypochlorite solution (1 %) for 5 min and thoroughly rinsed in distilled water. After that, seeds were germinated in a growth chamber at 24/21 °C and 16/8 h light/darkness conditions. After 3 days, germinated seeds were selected and carefully sown in a laminar flow chamber to minimize fungal contamination. Pots were maintained for 15 weeks (from May to July 2014) under greenhouse conditions.

### Fluorescence measurements

After 15 weeks and before *P. lanceolata* individuals were harvested, fluorescence parameters were evaluated. As we stated above, *P. lanceolata* is dependent on AMF, and therefore, we suspect that the absence of fungal relationship can be translated into a loss of photosynthetic efficiency. Chlorophyll *a* emission was monitored with a fluorescence imaging system (Imaging-PAM M-Series, Walz, Effeltrich, Germany). Five plants per treatment were kept in darkness for 5 min to allow all reaction centres to open and to minimize fluorescence associated with the energization of the thylakoid membrane (Krause *et al.* 1982). After this, the plants were successively illuminated at an intensity of 0.5 mol m^−2^ s^−1^ for a measurement of *F*_0_ (the minimum fluorescence of dark-adapted leaves), with a saturating pulse of intensity 2700 mol m^−2^ s^−1^ for measurement of *F*_m_ (the maximum fluorescence of dark-adapted leaves). With this procedure, we measured fluorescence parameters as PSII efficiency (*Ψ*_II_), regulated and non-regulated dissipated energy rate (*Ψ*_NPQ_ and *Ψ*_NO_, respectively), non-photochemical quenching rate (qN) and electronic transporting rate (ETR).

### Plant harvest

After the fluorescence measurement, the plants were gently removed from the pots and the roots of each plant were gently washed to remove any soil adhered, and then roots, shoots and hypocotyls were measured in length. Subsequently, the fresh weight of aerial and belowground material was measured and then plant material was dried in an oven at 70 °C for a minimum of 72 h to collect dry weights. Due to the key role of AMF in P uptake, we also measured the content of P in leaves and roots of *P. lanceolata* using ICP-OES (Perkin Elmer Optima 4300 DV).

Additionally, the root material from three plants of each pot was used for the measurements of root colonization. Roots from these plants were washed under tap water to remove soil particles. After that, roots were cut into small fragments (±1 cm), and these fragments were stained following the method of Walker (2005), slightly modified. The root fragments were briefly introduced into amber glass vials containing Coomassie Blue, the dye used for root staining, and covered with a cotton mesh (1 mm diameter) in order to prevent root loss during staining stages. The AMF colonization was assessed using a modified grid-line intersection method according to Lorenzo *et al.* (2013). To avoid misinterpretation, percentages of control, SA and SS were averaged and compared with non-sterile treatments since a slight percentage of colonization in sterilized treatments was found.

### Statistical analyses

To valorize the effect of *A. dealbata* on soil fungal structure, GelCompar II (Applied Maths, Belgium) was used in the cluster analysis of AMF based on the DGGE results. The unweighted pair-group method with arithmetic mean algorithm and the Pearson product–moment correlation coefficient were used for the analysis. Richness, defined as the number of species, was calculated as the total number of bands per sample. To calculate abundance and diversity, defined as the number of different species and their relative frequency, gel bands were classified according to their intensity in six categories. Diversity was calculated using a modification of the Shannon index, *H*′ = −Σ[(*n_i_*/*N*) Ln(*n_i_*/*N*)], where *n_i_* had one of four possible values (1–6), depending on band intensity. However, we assume that ecological parameters as defined here require a cautious interpretation since bands from DGGE cannot be unmistakably translated into AMF species (van Elsas and Boersma 2011).

The effect of the independent variables of the model (sterilization or not and species) on stem, hypocotyl and root length, aboveground and belowground biomass, P content, mycorrhizal colonization and fluorescence was evaluated using two-way analysis of variance (ANOVA). When interactions between independent variables were found, the effects were investigated through pairwise comparisons using Tukey's honest significant difference or Dunnett's T3 as the *post hoc* test. All data were previously subjected to Kolmogorov–Smirnov test for normality and Levene's test to check homoscedasticity of the variances. The statistical analyses were carried out using the SPSS v.19 (Chicago, IL) software for Windows.

## Results

### Soil characteristics and microbial community analysis

**Figure 1.**
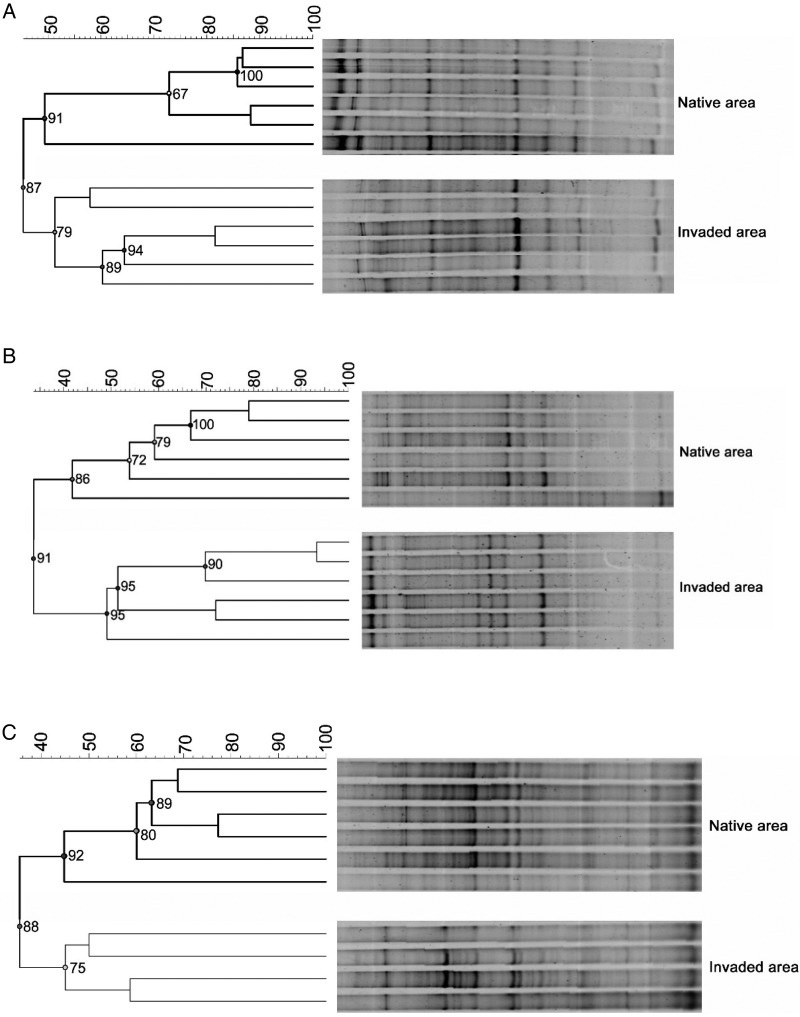
Dendrograms of AMF community structure based on PCR-DGGE bands, using the unweighted pair-group method with arithmetic mean algorithm and the Pearson product–moment correlation coefficient. S1 (A), S2 (B) and S3 (C) are the soils used in the assay.

Data from PCR-DGGE showed us alterations in the structure of soil AMF community. The cluster analyses revealed differences between the invaded and native zones in each shrubland (S1, S2 and S3), as indicated in the tree diagram (Fig. 1). In each native area studied, AMF community of invaded soils clustered together and separated from non-invaded samples. In contrast, AMF diversity, richness and density did not present significant differences between invaded and non-invaded areas (data not shown).

### Fluorescence measurements

Parameters related to fluorescence were significantly affected in *P. lanceolata* due to the treatments during the timing of the assay (Table 1). In general, we observed that plants with the non-sterile shrub inoculum presented higher photosynthetic activity, indicated as a significant increase in the effective quantum yield at PSII (*Ψ*_II_), or a reduction in the qN. While the ETR was reduced or virtually inhibited in other treatments, values of NSS were significantly increased.

**Table 1.** Values of the effective quantum yield at PSII (*Ψ*_II_), the quantum yield of light-induced heat dissipation (non-photochemical quenching, *Ψ*_NPQ_) and the quantum yield of all processes alternative to light-induced heat dissipation and photochemical use (*Ψ*_NO_). The ETR and the qN in *P. lanceolata* plants. Different letters indicate significant differences in Tukey's or Dunnett's T3 *post hoc* test at *P* ≤ 0.05 level.

	Control	NSA	SA	NSS	SS
*Ψ*_II_	0.004 (±0.01)**b**	0.012 (±0.02)**b**	0.005 (±0.01)**b**	0.14 (±0.05)**a**	0.022 (±0.02)**b**
*Ψ*_NPQ_	0.62 (±0.08)	0.66 (±0.06)	0.65 (±0.05)	0.61 (±0.05)	0.61 (±0.02)
*Ψ*_NO_	0.38 (±0.09)	0.33 (±0.06)	0.35 (±0.06)	0.26 (±0.01)	0.37 (±0.03)
ETR	<0.001 (±0.00)**b**	<0.001 (±0.00)**b**	2.02 (±2.58)**b**	28.77 (±8.41)**a**	4.45 (±4.01)**b**
qN	11.26 (±0.28)**ab**	10.92 (±0.5)**ab**	11.40 (±0.29)**a**	10.61 (±0.27)**b**	11.19 (±0.19)**ab**

### Plant growth and P content

We found a general effect of species and treatment in the two-way ANOVA in the growth and development of *P. lanceolata* (Table 2). Additionally, significant values of interaction in the model between the independent variables *species* × *treatment* were observed. Therefore, we can anticipate that differences found in the variables considered are not the same within species (*A. dealbata* or native species inoculum) than within treatments (sterilized or non-sterilized).

**Table 2.** Two-way ANOVA results, including independent variables (treatment and species) and interaction (T × S) for the leaf, hypocotyl and root length; dry weight of leaves and roots (aboveground and belowground biomass), P content in leaves and roots and percentage of AMF colonization in roots.

	df	Leaf length		Hypocotyl length		Root length		DW (leaves)		DW (roots)		*P* (leaves)		*P* (roots)		Colonization	
		*F*	*P*	*F*	*P*	*F*	*P*	*F*	*P*	*F*	*P*	*F*	*P*	*F*	*P*	*F*	*P*
Treatment	2	169.39	<0.001	2.66	0.154	0.56	0.484	1778.28	<0.001	13.27	<0.05	1250.57	<0.001	147.31	<0.001	22.42	<0.01
Species	2	193.28	<0.001	4.16	0.088	1.35	0.289	1312.97	<0.001	13.92	<0.05	1144.25	<0.001	58.55	<0.001	18.47	<0.01
T × S	4	219.10	<0.001	0.25	0.638	22.86	<0.01	1668.54	<0.001	23.99	<0.01	1293.86	<0.001	74.27	<0.001	21.06	<0.01

**Figure 2.**
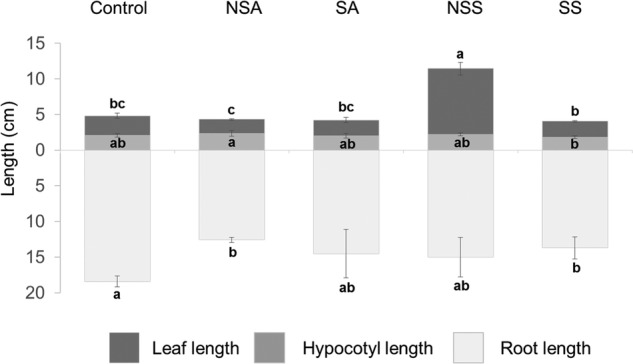
Length of root, leaf and hypocotyl in all treatments. NSA, non-sterilized acacia roots; SA, sterilized acacia roots; NSS, non-sterilized shrub roots and SS, sterilized shrub roots. Different letters indicate significant differences at *P* ≤ 0.05 level.

The two-way ANOVA showed that biometric parameters were affected by species and treatment. There were significant differences between treatments in length and biomass of *P. lanceolata*. In general, we found an overall increase in plant growth in pots containing non-sterile inoculum of native shrubs. More specifically, we found an evident increase in the length of *Plantago* leaves (*P*< 0.001) when plants were grown with non-sterile shrub inoculum (Fig. 2).

**Figure 3.**
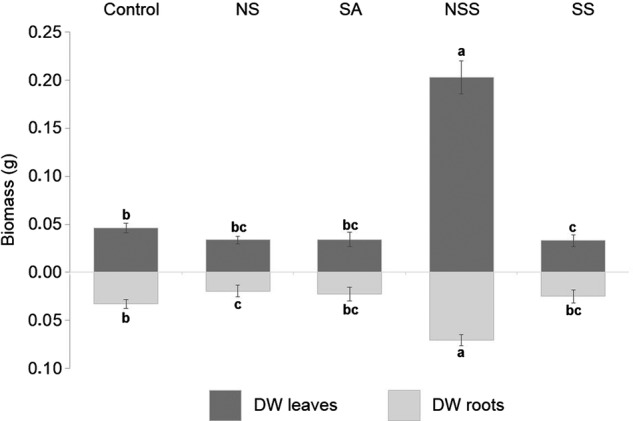
Dry weight (DW) of roots and leaves in all treatments. NSA, non-sterilized acacia roots; SA, sterilized acacia roots; NSS, non-sterilized shrub roots and SS, sterilized shrub roots. Different letters indicate significant differences at *P* ≤ 0.05 level.

**Figure 4.**
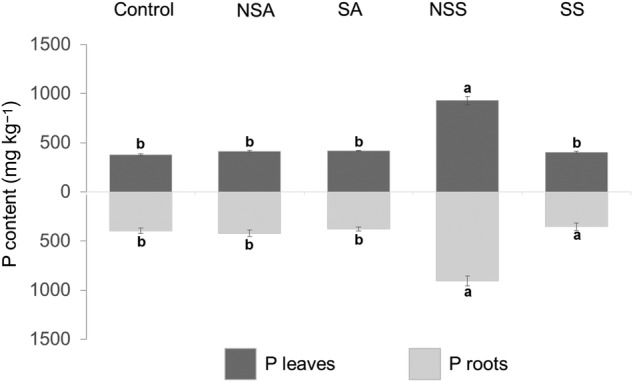
Phosphorus content of roots and leaves in all treatments. NSA, non-sterilized acacia roots; SA, sterilized acacia roots; NSS, non-sterilized shrub roots and SS, sterilized shrub roots. Different letters indicate significant differences at *P* ≤ 0.05 level.

As occurred with growth, the two-way ANOVA indicated that leaf and root biomass were affected by species, treatment and their interaction. Overall, the effect of NSS was particularly evident in biomass production (Fig. 3). When *P. lanceolata* grew with native shrub inoculum, we found a significant increase in the production of aerial biomass in comparison with control (341 %), NSA (500 %), SA (492 %) and SS (517 %; *P*< 0.001, in all cases). Complementarily, the production of root biomass in NSS was also significantly increased in comparison with control (110 %), NSA (263 %), SA (214 %) and SS treatment (184 %; *P*< 0.001 in all cases). A general increase in the P content related to NSS treatment in both the aerial and root content was also evident (Fig. 4).

### Mycorrhizal colonization

**Figure 5.**
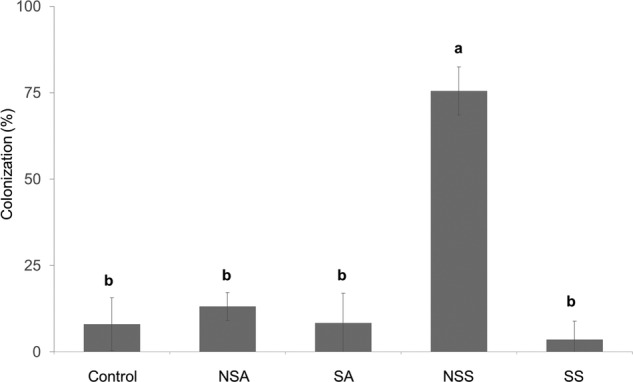
Percentage of colonization in *P. lanceolata* roots. NSA, non-sterilized acacia roots; SA, sterilized acacia roots; NSS, non-sterilized shrub roots and SS, sterilized shrub roots. Different letters indicate significant differences at *P* ≤ 0.05 level.

Significant differences (*P*< 0.001) were found between treatments in mycorrhizal colonization of *P. lanceolata* roots (Fig. 5). Plants treated with NSS inoculum had the highest colonization rate, 75 % of root surface colonized compared with the 13 % of colonization in NSA treatment. We found a slight percentage of colonization in treatments with sterile inoculum. Nevertheless, there were no differences in colonization rates between treatments with sterile roots and control.

## Discussion

As we hypothesized, cluster analysis from DGGE revealed that the structure of AMF community in non-invaded areas was different than community from areas with *A. dealbata* presence. In some cases, invasive plants have the capacity to disrupt mutualisms between AMF and native species (Klironomos 2003; Meinhardt and Gehring 2012), even decreasing the competitiveness of native plants (Yang *et al.* 2014). In this sense, it is interesting to note that previous studies indicated that *A. dealbata* does not seem to be benefited by specific associations with AMF (Crisóstomo 2012) and so the change in the AMF structure could be produced indirectly. Apparently, the low dependence on AM symbionts could be related with their highly invasive success (Pringle *et al*. 2009). Arbuscular mycorrhizal fungi are obligate biotrophs that need host plants in order to live (Smith and Read 1997), and the change in the dominant plant species in the ecosystem could unbalance the established equilibrium favouring some fungal species. Nevertheless, the reduction of plant community diversity that takes place in areas invaded by *A. dealbata* (Lorenzo *et al.* 2012) should decrease AMF community richness (Van der Heijden *et al.* 1998), which does not occur. We suggest that the change in plant composition and dominance in the structure of the aboveground ecosystem could be the main force that drives the change in AMF structure. The reduction in plant diversity under *A. dealbata* canopy (Lorenzo *et al.* 2012) decreases the possibilities of finding compatible mutualisms. Therefore, selective forces towards more competitive and adaptable fungal species are probably active, contributing to the explanation of the shift in the AMF community structure. In this sense, impact is the term usually employed to describe the effects produced after the entrance of the invasive plant (Hejda *et al.* 2009). Following interpretation of data from DGGE, we consider that the term ‘restructuration’ or ‘addressing’ can be more appropriate than ‘impact’ since the diversity and richness of AMF species were not affected in the invaded soils.

Native plants can vary widely in response to the AMF change produced by IAPs. It was observed that the inhibition of native mycorrhizas produced by the nonmycorrhizal IAP *Alliaria petiolata* modifies the scenario and reduces the competitive ability of native plants (Stinson *et al*. 2006). In the case of *A. dealbata*, the structural change produced in AMF in the invaded soils could not be related with the main hypothesis used to explain its invasive success (fast growth, sprouting, allelopathy), but with the low dependence on AM symbionts. Studied ecosystems are generally dominated by plants from different functional groups, as we found in agricultural fields, shrublands, grasslands or even forest dominated by non-nitrogen-fixing species (Lorenzo *et al.* 2012; Hernández *et al.* 2014; Souza-Alonso *et al.* 2014, 2015). Therefore, observed changes in AMF structure are produced by the obvious replacement in the identity of plant species dominance in areas invaded by *A. dealbata*. Nevertheless, it was also suggested that substitution in the main functional group can also be relevant in the response of the AMF community (Hoeksema *et al.* 2010; Lin *et al.* 2015).

Our second objective was to evaluate the change shown on AMF structure induced by *A. dealbata* on the growth and development of *P. lanceolata*. This species is highly colonized and dependent on AMF for development (Gange and West 1994; Ayres *et al.* 2006; De Deyn *et al.* 2009; Cotuna *et al.* 2013). Additionally, in terms of symbiotic relationships, *P. lanceolata* seems to be favoured by the coexistence with plant-related species (Bever 2002). Therefore, changes in the composition or structure of the established AMF communities probably entail consequences on plant development and growth.

Results from fluorescence measurements, mainly the significant increase in *Ψ*_II_ and ETR in plants growing with native roots inoculum, indicate that the new structure of AMF present in the invaded area produced a strong decline in photosynthetic efficiency, suggesting a possible damage to the photosynthetic machinery (Martínez-Peñalver *et al.* 2012) of *P. lanceolata*. This fact is supported by the external appearance of plants grown without native inoculum, presenting signs of chlorosis and nechrosis with evident tissue damage. A reduction in the amount of light energy–indicated by the increase in *Ψ*_II_—reaching the photosynthetic apparatus usually entails a significant decrease in the production of carbon-derived compounds (Genty *et al.* 1989; Rolfe and Scholes 1995; Li *et al.* 2008). Therefore, the damage in the photosynthetic apparatus contributes to the evident decrease in *P. lanceolata* growth.

Plants grown in pots with NSS inoculum presented maximum growth, biomass, P content and colonization. This occurs because AMF establish mutualistic associations with host plants, which raises the nutrient uptake, mainly P (Jeffries *et al.* 2003; Hassan *et al.* 2013). Additionally, it is important to consider that the difference found in AMF structure in invaded soils does not necessarily correspond to AMF species that effectively infect *P. lanceolata* roots. Instead of the general trend that indicates that AMF do not present high host specificity and plants are able to relate to almost every AMF (Giovannetti and Hepper 1985; Zhang *et al.* 2010), our results, surprisingly, do not go along the same line. We found different fungal composition, whereas density, diversity and richness values were not significantly altered. Regardless of the change in the ‘AMF species identity’—despite not being specifically addressed—we assume that the presence of propagules, which grants opportunities to establish plant–fungal relationships, was similar between invaded and non-invaded areas. Therefore, we should expect that the number of infections were similar, but this was not the case. Consequently, we cautiously suggest that, in some cases, *P. lanceolata* presents some level of specificity in selecting their partners, or vice versa, probably influenced by nutrient requirements, soil environmental conditions or the absence of local adaptation. Another plausible explanation could be the different form of AMF propagules identified in DGGE analyses. The composition of AMF community is important, but the form in which the AMF species is present—in the form of hyphae or spores—could also be relevant.

Differences found in root colonization can be related to the soil environment produced by *A. dealbata* in the field. Root–AMF association is a chemically modulated process and AMF can sense components of the rhizosphere (Harrison 2005 and references therein). The extent of the rhizomatous system of *A. dealbata* produces severe physico-chemical changes in soils under its canopy (Lorenzo *et al.* 2010; Souza-Alonso *et al.* 2014), producing an unfavourable ambient in which the association of AMF with plant roots can be challenging. These difficulties can be produced mainly at two levels: diminishing spore germination or limiting the growth of the hyphal tube in the search of a host root. In this sense, the chemoactive compounds that this species releases (Reigosa *et al.* 1999; Lorenzo *et al.* 2010, 2011; Aguilera *et al.* 2015) do not seem to affect native AMF colonization of *P. lanceolata* in field conditions (Lorenzo *et al.* 2013).

Structural changes produced in the AMF community in invaded soils could also have further consequences. Native plants and mycorrhizal fungal communities show interdependence, and so reassembly of one community may be limited by the reassembly of the other (Lankau *et al.* 2014). It has previously been indicated that the influence of invasive species on soil characteristics remains even after their removal, an effect known as ‘legacy effect’. Residual effects are usually related to the age (short- or long-term invasion) and degree of invasion (low or high level of invasion) and takes place at several levels: soil nutrient changes (Marchante *et al.* 2008), organic matter content (Novoa *et al.* 2013) and even those that alter the AMF community (Lankau *et al.* 2014; Shannon *et al.* 2014). Therefore, it should be noted that the modification in the AMF structure could condition individual plant establishment after *A. dealbata* management, complicating ecosystem restoration processes.

## Conclusions

Our results indicate that *A. dealbata* effectively changes the structure of the AMF community in the invaded shrublands with negative consequences. The change in the identity of AMF species constrained the growth of plants that depends on AMF, such as *P. lanceolata*. Our work highlights the importance of maintaining soil communities, particularly in regards to the entrance of invasive dominant species.

## Sources of Funding

This work was supported by The Agroalimentary Research Center (CIA) of the Regional Government of Galicia, also A.G.-C. was awarded by the PhD Student grant of University of Vigo (00VI 131H 641.02). This is a contribution from the Alien Species Network (Ref. R2014/036 – Xunta de Galicia, Regional Government of Galicia).

## Contributions by the Authors

P.S.-A., L.G. and A.G.-C. conceived and designed the idea. A.G.-C. and P.S.-A collected the data. A.G.-C. and P.S.-A. ran the statistics. A.G.-C., P.S.-A. and L.G. discussed the results and wrote the manuscript.

## Conflict of Interest Statement

None declared.
